# Microbial Communities in Biocrusts Are Recruited From the Neighboring Sand at Coastal Dunes Along the Baltic Sea

**DOI:** 10.3389/fmicb.2022.859447

**Published:** 2022-06-16

**Authors:** Karin Glaser, Ahn Tu Van, Ekaterina Pushkareva, Israel Barrantes, Ulf Karsten

**Affiliations:** ^1^Department of Applied Ecology and Phycology, Institute of Biological Sciences, University of Rostock, Rostock, Germany; ^2^Department of Biology, Botanical Institute, University of Cologne, Cologne, Germany; ^3^Research Group Translational Bioinformatics, Institute for Biostatistics and Informatics in Medicine and Ageing Research, Rostock University Medical Center, Rostock, Germany

**Keywords:** coastal sand dune, Bacteria, Fungi, microalgae, cyanobacteria, network, biocrust, high-throughput sequencing

## Abstract

Biological soil crusts occur worldwide as pioneer communities stabilizing the soil surface. In coastal primary sand dunes, vascular plants cannot sustain due to scarce nutrients and the low-water-holding capacity of the sand sediment. Thus, besides planted dune grass, biocrusts are the only vegetation there. Although biocrusts can reach high coverage rates in coastal sand dunes, studies about their biodiversity are rare. Here, we present a comprehensive overview of the biodiversity of microorganisms in such biocrusts and the neighboring sand from sampling sites along the Baltic Sea coast. The biodiversity of Bacteria, Cyanobacteria, Fungi, and other microbial Eukaryota were assessed using high-throughput sequencing (HTS) with a mixture of universal and group-specific primers. The results showed that the biocrusts recruit their microorganisms mainly from the neighboring sand rather than supporting a universal biocrust microbiome. Although in biocrusts the taxa richness was lower than in sand, five times more co-occurrences were identified using network analysis. This study showed that by comparing neighboring bare surface substrates with biocrusts holds the potential to better understand biocrust development. In addition, the target sequencing approach helps outline potential biotic interactions between different microorganisms groups and identify key players during biocrust development.

## Introduction

Sand dunes are the first line of physical defense against the sea at many natural coasts. Dunes are unique ecosystems in the transition zone between terrestrial and marine environments, where interactions between geology, climate, and vegetation create highly dynamic environments (Miller et al., 2010). Growth of coastal dunes depends on sand supply and stability, which is influenced by biotic (vegetation cover) and abiotic factors (wind, waves, and precipitation). Coastal dune systems are harsh environments for vascular plants due to a wide variety of environmental stressors such as strong wind, substrate mobility, scarcity of nutrients, and soil water, occasionally extremely high temperatures near surface, intense radiation, flooding, and salt spray (Miller et al., 2010). Under these conditions, the growth and development of vascular plants are limited (except for anthropogenically planted beach grass) and only specialized communities of organisms such as biological soil crusts (biocrusts) can establish (Schulz et al., 2016).

Biocrusts are formed by living organisms and their by-products, creating a top-soil layer of inorganic particles bound together by sticky organic compounds. Macroscopic Lichens and Bryophytes, Cyanobacteria, and Algae represent the most important phototrophic components in biocrusts and live in intimate association with heterotrophic organisms like Bacteria, Archaea, Fungi, and Protists (Elbert et al., 2012). Biocrusts occur on all continents on Earth, in arid and semi-arid hot habitats, as well as in other climatic zones, where soil moisture is limiting and higher plant cover is sparse (Belnap et al., 2001). In temperate regions, these habitats include sandy coastal and inland dunes, disturbed areas (forest wind breakage, fires, etc.), or barren soil. Biocrusts generally occupy soil spaces free of vascular plants, and thus can represent up to 70% of the living cover (Kern et al., 2019). They can be characterized as “ecosystem-engineers” forming water-stable aggregates that have important, multifunctional ecological roles in primary production, nitrogen (N) cycling, mineralization, water retention, and stabilization of soils and dust trapping (Evans and Johansen, 1999; Reynolds et al., 2001; Lewis, 2007; Castillo-Monroy et al., 2010). A review of these microbiotic crusts clearly indicates the important ecological role of these communities for global carbon (C) fixation (*ca*. 7% of terrestrial vegetation) and nitrogen (N) fixation (about 50% of terrestrial biological N fixation) (Elbert et al., 2012).

While extensive data exist on the biology, ecology, biogeochemistry, and disturbance of biocrusts in arid and semi-arid regions from all over the world (Belnap et al., 2001; Weber et al., 2016), much less is known about such pioneer communities from temperate regions (Corbin and Thiet, 2020). Temperate biocrusts support a highly diverse photoautotrophic community (Glaser et al., 2018; Mikhailyuk et al., 2019). Large filamentous Cyanobacteria, as well as filamentous green algae, are especially important for the development of biocrusts because their filaments and sticky mucilaginous sheaths glue soil particles together and form a stable matrix in the top soil. In temperate regions, biocrusts are abundant at highly disturbed sites especially on sandy soils and can dominate dune ecosystems (Dümig et al., 2014; Kidron and Büdel, 2014; Gypser et al., 2016; Fernández-Alonso et al., 2021; Rieser et al., 2021). Nevertheless, up to now only a few studies focused on biocrusts from coastal dunes and described the biodiversity of Algae, Cyanobacteria, and Protists (Schulz et al., 2016; Mikhailyuk et al., 2019; Roshan et al., 2020; Fernández-Alonso et al., 2021).

As microbial biomass and activity are higher in biocrusts compared to bulk soil (Ngosong et al., 2020; Fernández-Alonso et al., 2021), it is reasonable to assume that there are more biotic interactions between microorganisms in biocrusts compared to bulk soil (Pombubpa et al., 2020). Studies that compare co-occurrences in biocrusts across domains are rare (Pombubpa et al., 2020; Glaser et al., 2022). Such studies can uncover so far unknown biotic interactions between microbial groups, resulting in a better understanding of biocrust microbiomes and key connectors. One example of such potential interactions is a study of Cercozoa in biocrusts, where the authors observed an unexpectedly high abundance of algi- and eukaryvore species besides the more typical bacterivores, indicating a higher importance of predator-prey relationships between Cercozoa and phototrophic microorganisms (Roshan et al., 2020).

This study aims to describe the microbial biodiversity in biocrusts of coastal sand dunes along the Baltic Sea compared to the neighboring biocrust-free sands. In addition, co-occurrences as a hint toward potential biotic interactions of microorganisms from different domains were identified in these microecosystems. We hypothesize that microorganisms interact more closely in biocrusts compared to bare sand, which should be reflected by more inter- and intra-domain co-occurrences. Further, we hypothesize that the biocrust community is a subset of microbial organisms from the neighboring sand, but with a significant shift toward phototrophic microorganisms as key players.

## Materials and Methods

### Sampling Sites

**Figure 1:**
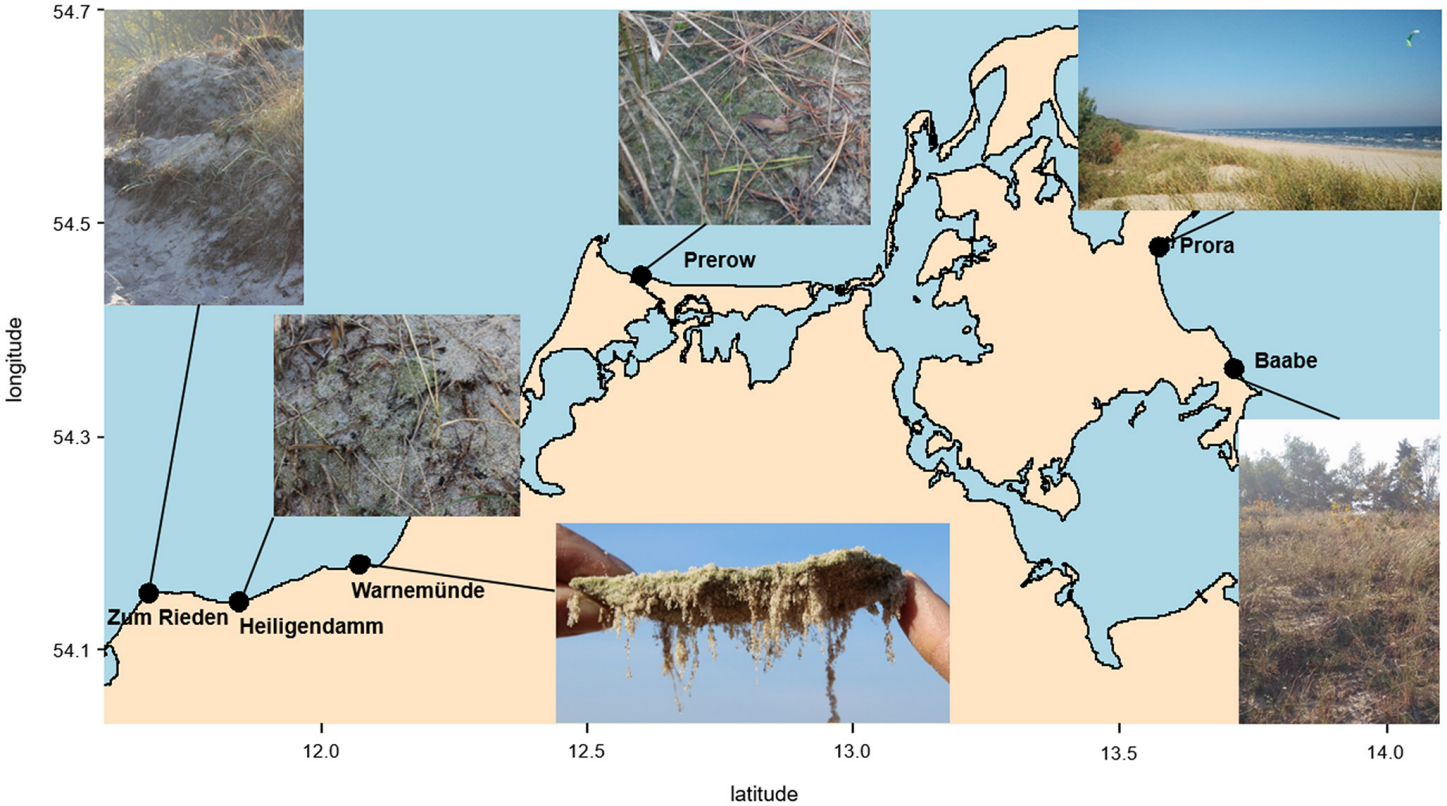
Map of northeast German coast, including the sampling sites and impressions of the biocrusts in dunes.

Sampling was conducted in October 2018 within two consecutive days. Six stations along the German coastline of the Baltic Sea were visited (Figure 1). The Baltic Sea is characterized by a distinct salinity gradient from East to West (Zettler et al., 2007) with 15–20 S_A_ at Riedensee and 5–10 S_A_ in Baabe. At each location, human activity was observed due to frequent usage of the beaches. Biocrusts, as well as neighboring biocrust-free sand, were collected from yellow dunes facing the sea, which were stabilized with planted dune grass (*Ammophila arenaria*). The surface soil with a maximum depth of 5 mm was collected as biocrust-free soil, which equals the thickness of the biocrusts. Samples from biocrusts, as well as neighboring sand, were collected by pooling three spots of surface soil, which was taken using a cork borer with 5 mm diameter. Samples were immediately frozen in the field for DNA extraction.

### Physicochemical Parameters

For the chemical analyses, biocrusts, as well as neighboring sand, were dried in the oven at 45°C for 24 h. Gravimetric water content was estimated after drying the samples for 24 h at 105°C. Electrical conductivity (EC) was determined by mixing 10 g air-dried sieved soil (<2 mm) samples with 50 ml deionized water (<5 μS/cm). After shaking for 1 h followed by 30 min of sedimentation, the EC of the supernatant was measured with an EC meter (Seven MultiTM, Mettler Toledo, Schwarzenbach/Germany, in Lab 731 probe). Total phosphorous (TP) was measured photometrically following a published protocol (Berthold et al., 2019). All measurements were performed in triplicates. Limit of detection was 0.091 μmol/L and limit of quantification was 0.272 μmol/L.

### DNA Extraction and Sequencing

Total DNA was extracted from 250 mg of each biocrust and each neighboring biocrust-free sand using DNeasy PowerSoil Kit (QIAGEN, Hilden, Germany) according to the instructions of the manufacturer. DNA content was quantified using QuBit 3.0 Fluorometer (Thermo Fisher Scientific, Waltham, MA, USA) with the high-sensitivity dye according to the protocol of the manufacturer.

Total DNA was sent to Microsynth AG (Balgach, Switzerland), where PCR and sequencing were performed using the Illumina MiSeq platform (v3, 2_300 bp). Four groups of microorganisms were targeted in the amplification step, namely, Bacteria (V3–V4 region of the SSU rRNA gene), Cyanobacteria (V4 region of SSU 16S rRNA gene), Eukaryota (V4 region of the SSU rRNA gene), and Fungi [internal transcribed spacer region (ITS) between small and large subunit]. The primers used in this study are presented in Supplementary Table 1. The raw sequencing data were deposited in the European Nucleotide Archive (ENA) under the project PRJEB48634.

### Quality Control and Assembly

All amplicons were reconstructed from the Illumina sequencing runs using the *pandaseq* program (version 2.11; Masella et al., 2012). Operational taxonomic units (OTUs) were then identified from these amplicons with USEARCH (version 6.1.544; Edgar, 2010), called from the *pick_open_reference_otus.py* script of QIIME 1.9.1 (Caporaso et al., 2010). The following databases were used as a reference for taxonomic assignments: 16S rRNA Greengenes (version 13.8; (McDonald et al., 2012) for Bacteria and Cyanobacteria; SILVA (version 132; Quast et al., 2013) for Eukaryota; and UNITE (version 12_11; Abarenkov et al., 2010) for Fungi. In all cases, a cutoff of 97% identity was applied. Then, OTUs with low confidence were excluded *via* the *remove_low_confidence_otus.py* script (Comeau et al., 2017). Furthermore, contaminant OTUs, such as those classified as chloroplasts, mitochondria, or non-bacterial, were removed from the bacterial dataset; and likewise, bacterial and chloroplasts sequences were removed from the Cyanobacteria data. Similarly, prokaryotic OTUs were removed from the Eukaryota dataset.

### Statistical Analyses

All statistical analyses were done in R (Version 4.1.1; R Development Core Team, 2009). Sequencing data were processed with the packages *phyloseq, vegan, AncomBC, metacoder*, and *circlize* (McMurdie and Holmes, 2013; Gu et al., 2014; Lin and Peddada, 2020; Oksanen et al., 2020). We obtained 18,190 ± 970 reads for Bacteria, 42,737 ± 1,947 reads for Cyanobacteria, 25,415 ± 1,829 reads for Fungi, and 43,417 ± 520 reads for Eukaryota per sample. Differences between alpha diversity were calculated using Welch's *t*-test. Ancom (analysis of compositions of microbiomes) with bias correction was used to measure significant differences in the relative abundance of OTUs. PerMANOVA (using the *vegan* function *adonis*) and nMDS (non-metric multidimensional scaling) analyses were performed based on the relative abundance using the Bray-Curtis dissimilarity index. Differential abundance of taxa and higher phylogenetic ranks were visualized using the function heat_tree (package: metacoder); differential abundance was calculated as log2 ratio between the median comparing the relative abundance between biocrust and sand samples of the respective phylogenetic rank. Network analyses were conducted using plot_net command from the phyloseq package: Cyanobacteria and Fungi are depicted in different color from the bacterial and eukaryotic dataset since these groups were targeted with specific primers. For visualization, chordDiagram from package circlize was used. Network analyses were based on Euclidean distance with a maximum distance of 0.1.

## Results

Biocrusts at an early stage were observed at all six sampling sites, as well as bare sand in proximity. Based on the macroscopic impression, we categorized the biocrusts as green algae dominated. The average DNA content was two times higher in biocrusts (average 6.6 ± 2.8 μg/ml) than in the adjacent sands (average 3.6 ± 3.1 μg/ml).

### Abiotic Parameters

Biocrusts and neighboring sands showed a generally low moisture content (<4%), but biocrusts exhibited higher water content than sand samples, although this difference was not significant (Table 1). The EC showed a significant difference between biocrusts (42 μS/cm) and sand samples (20 μS/cm; *p* < 0.01). Total phosphorus content was similar between biocrust and sand samples (~125 mg/g DW).

**Table 1:** Abiotic parameters of the sampling sites in biocrust and sand samples.

**Site**	**Biocrust**			**Neighboring sand**		
	**Moisture [%]**	**EC [μS/cm]**	**μg TP/mg DW**	**Moisture [%]**	**EC [μS/cm]**	**μg TP/mg DW**
Zum Rieden	0.63	63	127.3	0.39	22	92.91
Heiligendamm	0.66	24.6	63.82	0.64	12.06	69.68
Warnemünde	3.54	38.1	101.1	1.28	21.1	145.43
Prerow	0.15	43.8	137.2	0.11	24.5	219.35
Baabe	2.84	20.6	167.99	0.62	15.75	103.46
Prora	2.21	60.8	163.94	0.51	25.3	108

### Alpha-Diversity

**Figure 2:**
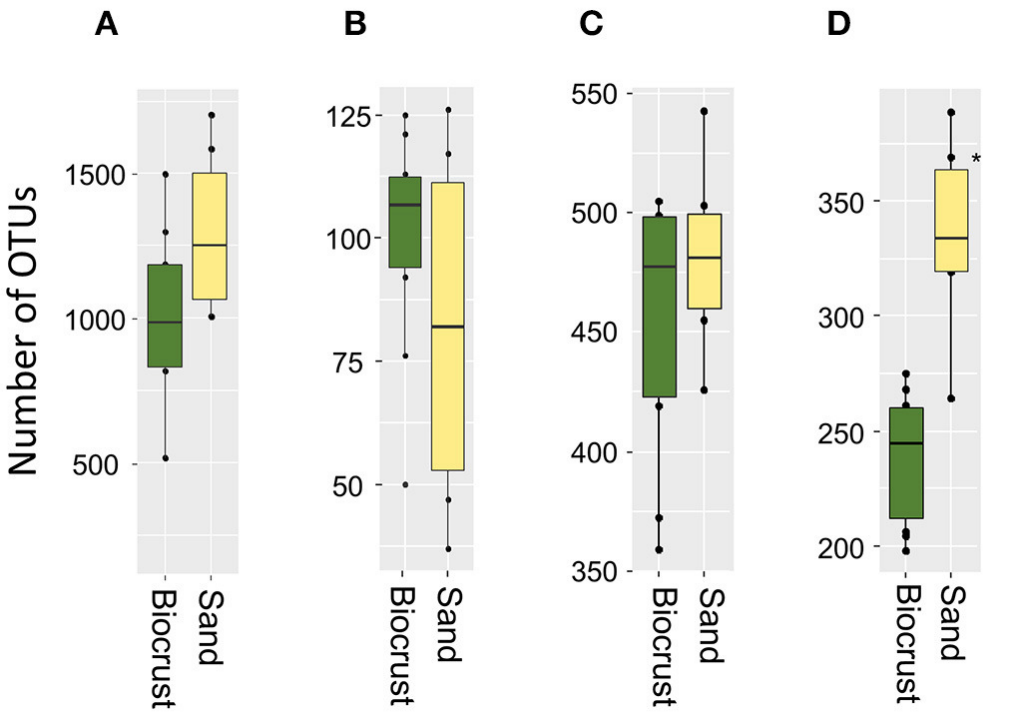
Richness as numbers of OTUs from biocrust and neighboring sand samples for each group: Bacteria **(A)**, Cyanobacteria **(B)**, Fungi **(C)**, Eukaryota **(D)**; differences were significant (*p < 0.05) in the fungal dataset.

The richness of Bacteria, Cyanobacteria, and Eukaryota was not significantly different between biocrusts and sand; nevertheless, 1.3 times more bacterial OTUs were observed in sand than in biocrusts (Figure 2). For Fungi, significantly more OTUs were detected in the sand (335 OTUs) than in the biocrusts (239 OTUs, *p*-value = 0.002; Figure 2).

**Figure 3:**
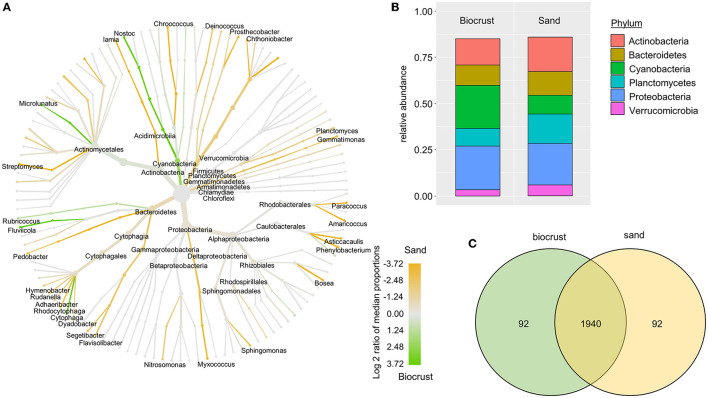
Analysis of bacterial OTUs comparing biocrust and neighboring sand samples; **(A)** taxonomic composition of the bacterial community: relative abundance of each clade was compared between biocrust and sand samples (log2 ratio between the median) and indicated with colors. Color code is given below the figure; **(B)** relative abundance of bacterial phyla (abundance ≥ 5%) compared between biocrust and sand samples; **(C)** venn diagram shows the number of shared and unique OTUs of biocrust and sand samples.

The most abundant bacterial phyla were Proteobacteria, Actinobacteria, Planctomycetes, and Bacteroidetes across all biocrust and sand samples (Figure 3B). The relative abundance of Cyanobacteria was 13% for the sand samples and 31% for biocrusts (although not significantly different, *p*-value = 0.14). Except for Cyanobacteria, all other bacterial phyla had a higher relative abundance in the sand than in the biocrusts (Figure 3A). In detail, seven cyanobacterial OTUs were significantly more abundant in biocrusts than in sand (Ancom, *p*-value < 0.05), with three OTUs belonging to *Leptolyngbya* spp. (~45 times more abundant in biocrusts) and four OTUs belonging to *Nostoc* spp. (~8 times more abundant). Overall, most of the OTUs (>90%) were shared between biocrust and sand samples with only a few OTUs (each <5%) detected in one habitat (Figure 3C).

**Figure 4:**
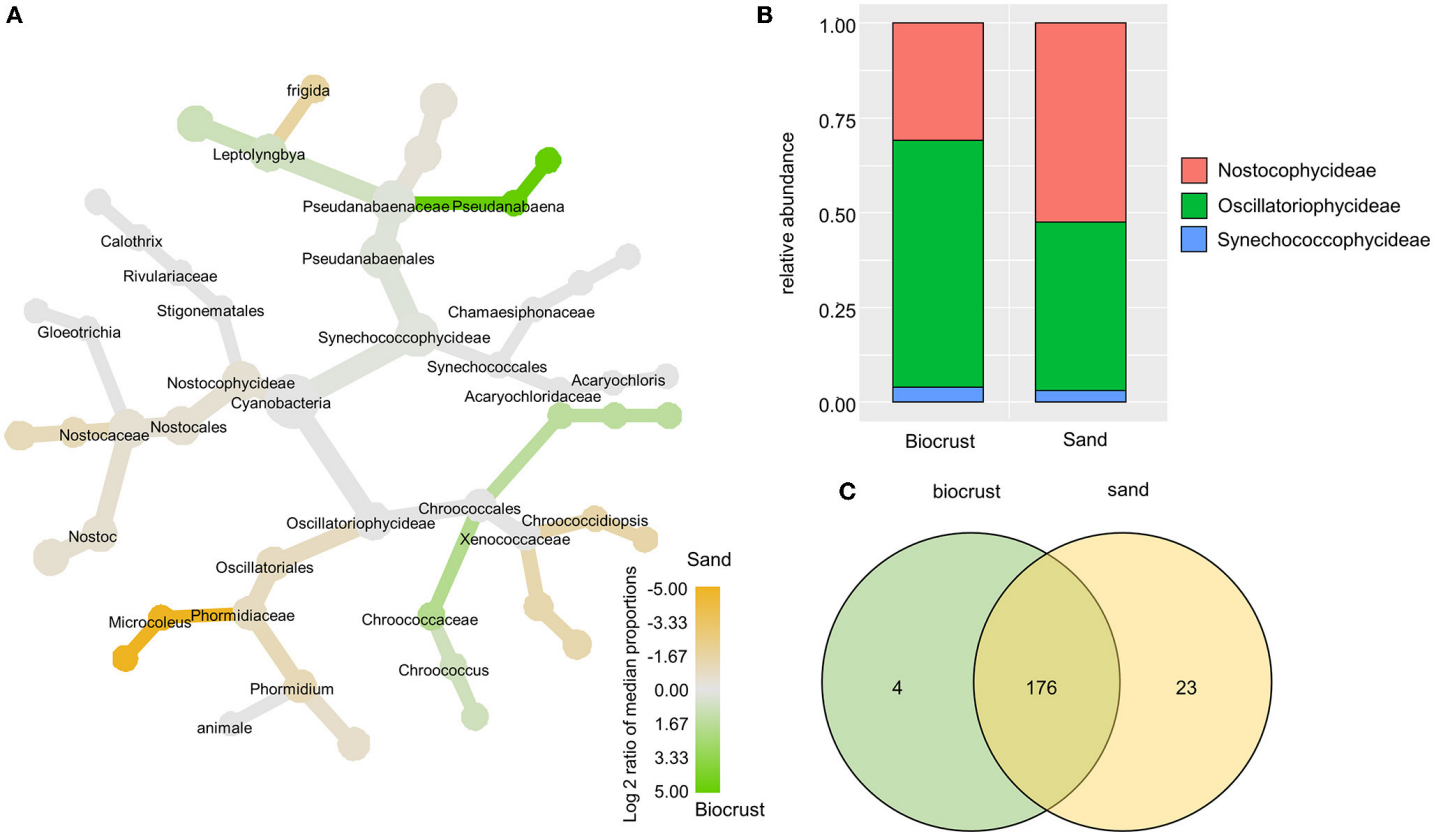
Analysis of cyanobacterial OTUs comparing biocrust and neighboring sand samples; **(A)** taxonomic composition of the cyanobacterial community: relative abundance of each clade was compared between biocrust and sand samples (log2 ratio between the median) and indicated with colors. Color code is given below the figure; **(B)** relative abundance of cyanobacteria classes compared between biocrust and sand samples; **(C)** venn diagram shows the number of shared and unique OTUs of biocrust and sand samples.

In the dataset obtained with cyanobacteria-specific primers, the majority of Cyanobacteria belonged to the class Oscillatoriophycideae (65 and 44% of total cyanobacterial reads in biocrust and sand samples, respectively) and the class Nostocophycideae (31 and 52% of total cyanobacterial reads in biocrust and sand samples, respectively). Members of the class Synechococcophycideae made up 3–4% of all OTUs (Figures 4A,B). In addition, *Nostoc* was the most abundant genus with ~56% relative abundance in both habitats represented by 33 OTUs. In general, most OTUs (87%) were shared between biocrust and sand samples with only a few OTUs solely detected in one habitat (Figure 4C).

**Figure 5:**
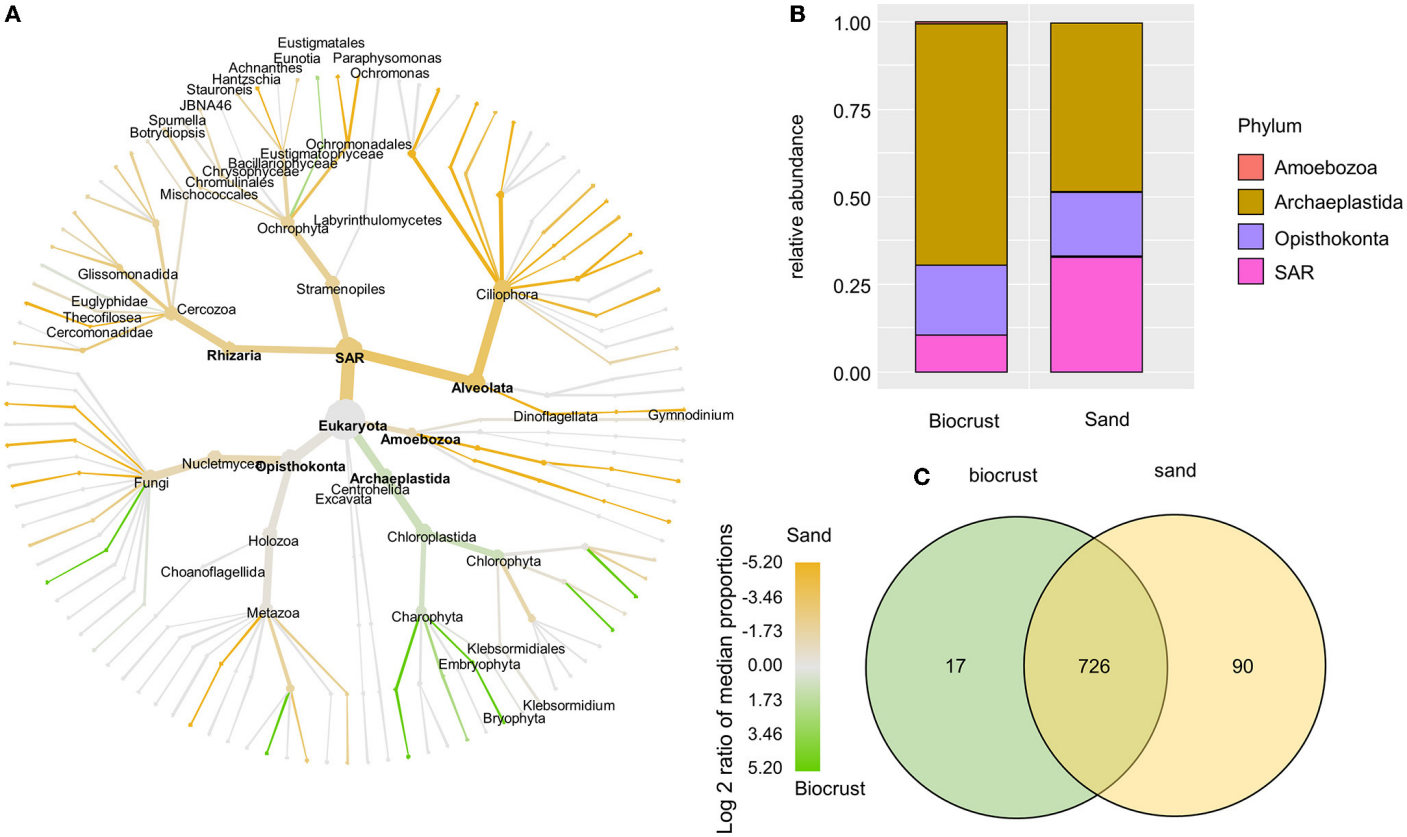
Analysis of eukaryotic OTUs comparing biocrust and neighboring sand samples; **(A)** taxonomic composition of the eukaryotic community: relative abundance of each clade was compared between biocrust and sand samples (log2 ratio between the median) and indicated with colors. Color code is given below the figure; **(B)** relative abundance of eukaryotic phyla compared between biocrust and sand samples; **(C)** venn diagram shows the number of shared and unique OTUs of biocrust and sand samples.

Most of the eukaryotic OTUs were assigned to the phyla Archaeplastida, Opisthokonta, and SAR (Figure 5B). In sand samples, the SAR supergroup had a significantly higher share than in biocrusts (33 and 10.5% of total eukaryotic reads in sand and biocrusts, respectively; Wilcoxon *p*-value = 0.016). *Vice versa*, Archaeplastida prevailed in biocrusts in comparison with sand, although this observation was not significant (47 and 67% of total eukaryotic reads in sand and biocrusts, respectively). Most of the phylogenetic lineages within the Archaeplastida were more abundant in biocrusts than in the sand (Figure 5A). *Vice versa*, most protozoa lineages, like Amoebozoa, Ciliophora, and Cercozoa, were 3–10 times more frequently detected in sand than in biocrusts. Within the Archaeplastida, the Bryophyta had an ~35 times higher relative abundance in biocrusts compared to sand (*p* < 0.035). The genus *Klebsormidium* (Archaeplastida, Charophyta) was assigned to 69 OTUs and made up nearly half of all OTUs in each sample (mean 42% of total eukaryotic reads). In general, most OTUs (87%) were shared between biocrust and sand samples (Figure 5C).

**Figure 6:**
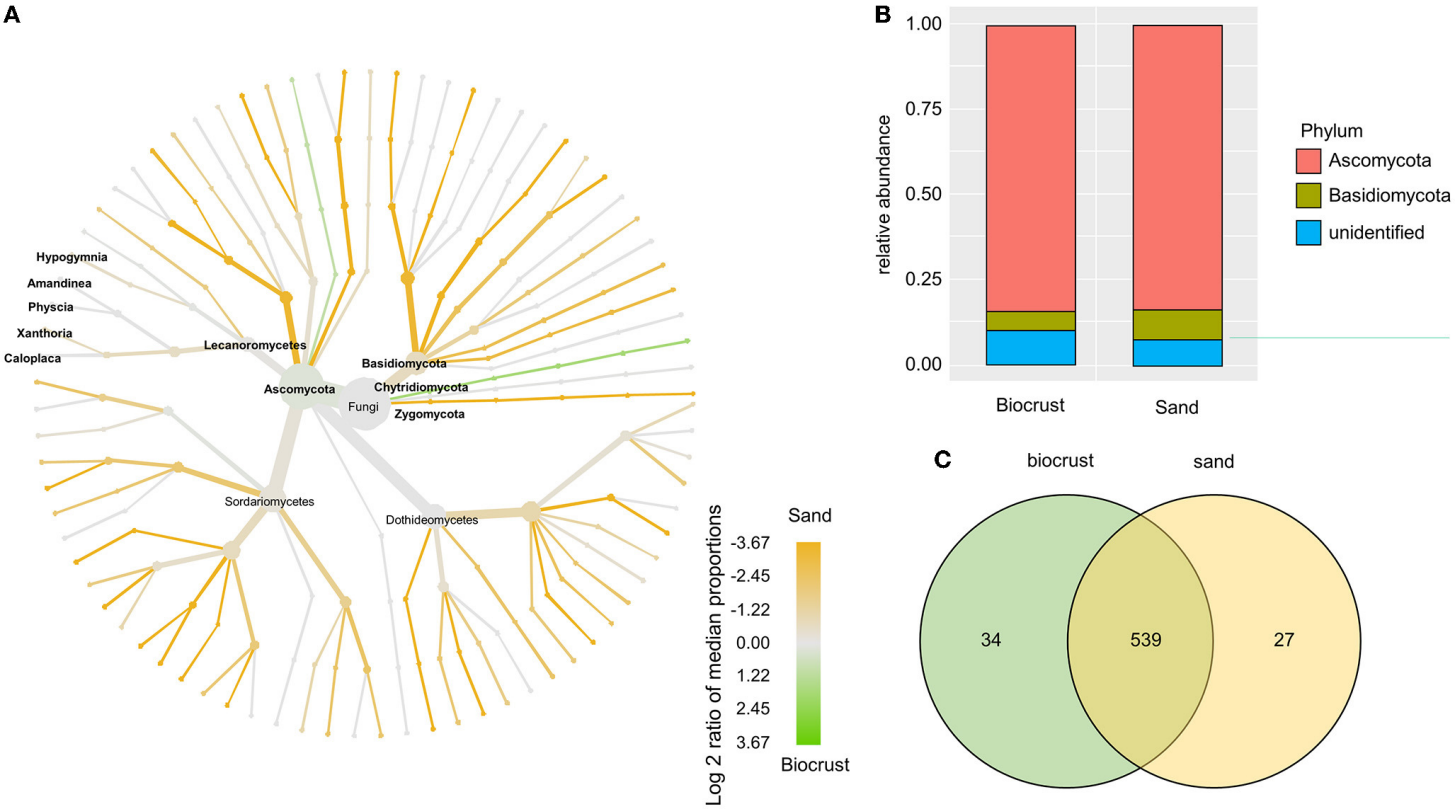
Analysis of fungal OTUs comparing biocrust and neighboring sand samples; **(A)** taxonomic composition of the fungal community: relative abundance of each clade was compared between biocrust and sand samples (log2 ratio between the median) and indicated with colors. Color code is given below the figure; **(B)** relative abundance of fungi classes compared between biocrust and sand samples; **(C)** venn diagram shows the number of shared and unique OTUs of biocrust and sand samples.

The fungal OTUs were assigned to the phylum Ascomycota (~84% in both habitats) and Basidiomycota (~6% in biocrusts, 9% in sand) (Figure 6B). The majority of fungal lineages were more abundant in the sand compared to biocrusts (Figure 6A). Five genera of lichenizing Fungi were identified with no significant differences in abundance between the two habitats, namely, *Amandinea, Caloplaca, Hypogymnia, Physcia*, and *Xanthoria*. In general, most OTUs (90%) were shared between biocrust and sand samples with only a few OTUs (~5 % each) solely detected in one habitat (Figure 6C).

### Beta-Diversity

**Figure 7:**
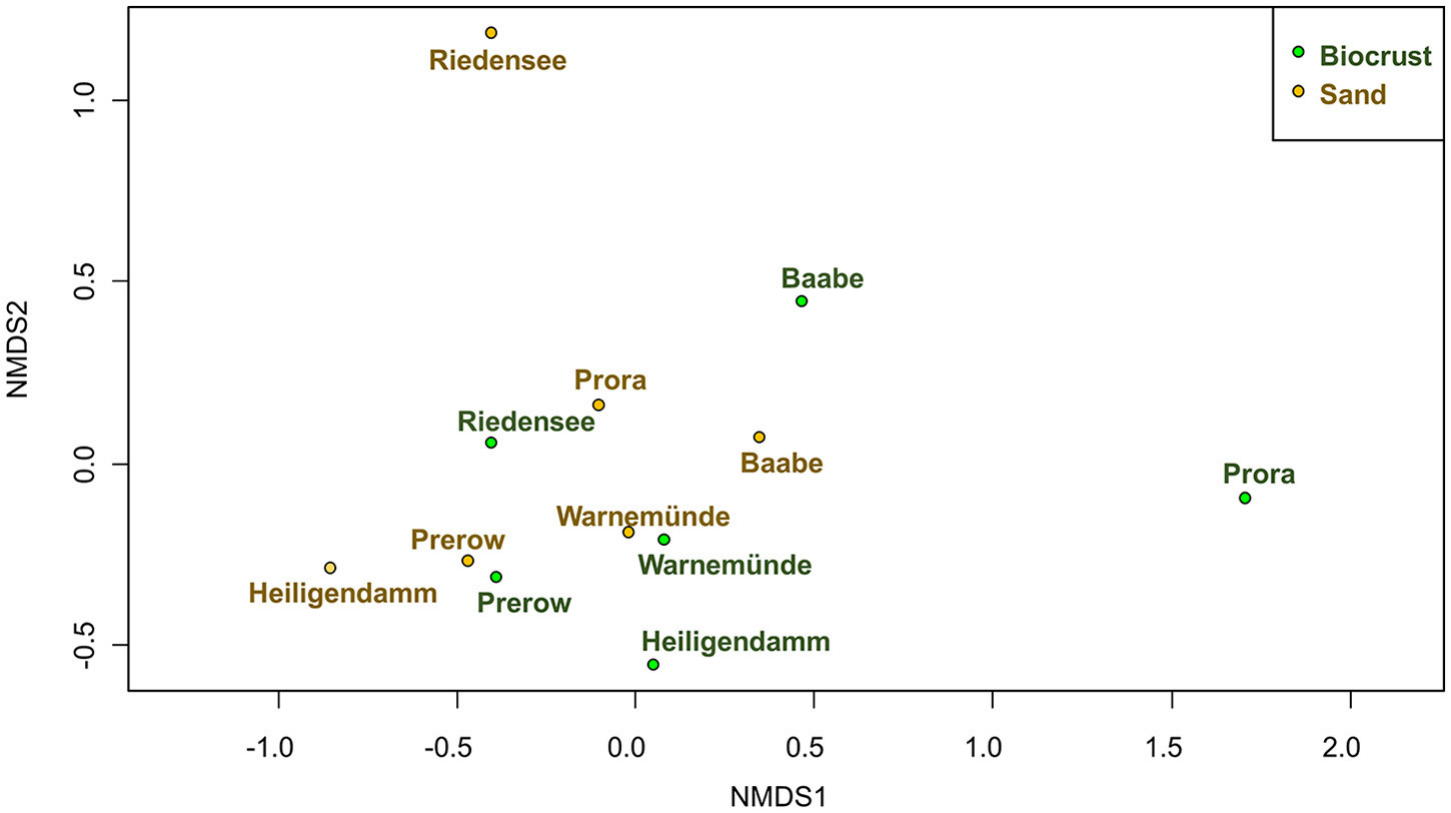
Community composition of microorganisms in biocrusts (green) compared to neighboring sand (brown) visualized using nMDS; stress = 0.11.

Community composition of biocrusts was significantly different from neighboring sand (PerMANOVA, 9.8% explained variance, *p* = 0.047; Figure 7). The most significant abiotic factor influencing community composition was the sampling site (PerMANOVA, 53.9% explained variance, *p* = 0.001). Therefore, biocrusts and sand samples from the same site were more similar to each other than two biocrust samples from different sites. However, abiotic factors, moisture, electronic conductivity, and total P (Table 1) were not significantly correlated with the community composition (PerMANOVA).

### Network Analysis

**Figure 8:**
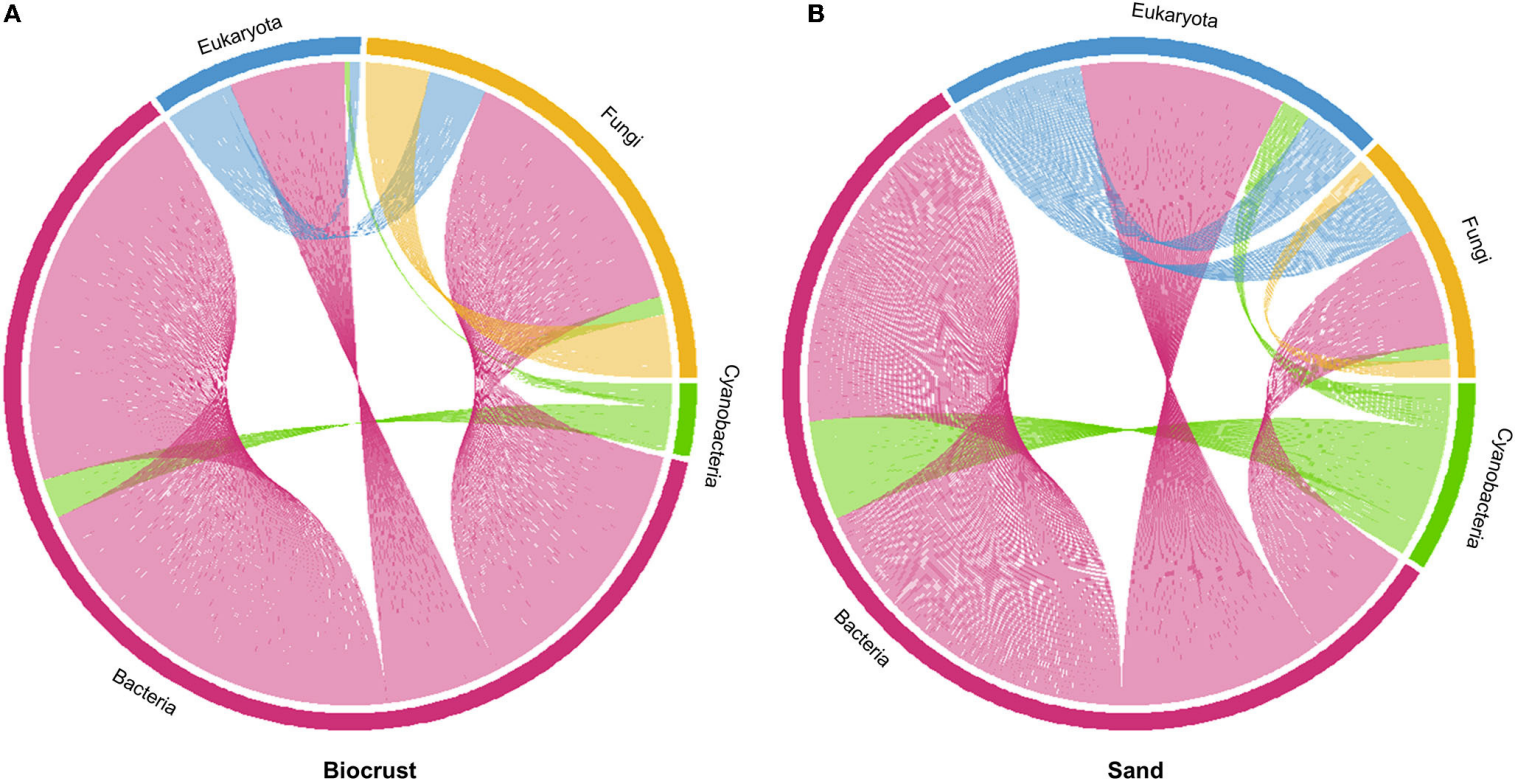
Co-occurrence networks of Bacteria (excluding Cyanobacteria), Cyanobacteria, Fungi, and Eukaryota (excluding Fungi); **(A)** biocrust with 2,072 connections; **(B)** sand with 506 connections.

Co-occurrence networks calculated on the entire dataset, including Bacteria, Cyanobacteria, Fungi, and Eukarya, showed that the biocrust network exhibited 2,072 connections among OTUs from different domains, while sand had only 506 connections (Figure 8).

In biocrusts, most connections were observed in the bacterial domain (1,506, excluding Cyanobacteria), followed by Fungi (253), Eukaryota (233, excluding Fungi), and Cyanobacteria (80), respectively (Figure 8A). Most connections of Bacteria were within the bacterial domain. Fungi were mostly connected with bacterial and other fungal OTUs. Only a few connections were observed between Cyanobacteria and Eukaryota.

In sand, most connections were also observed within the bacterial domain (308, Figure 8B). In contrast to biocrusts, the second most abundant connections were observed in Eukaryota (113), followed by Cyanobacteria (49) and Fungi (36). As seen for biocrusts, most of the bacterial connections were intra-domain. In contrast to the biocrusts, many co-occurrences were observed between Bacteria and Eukaryota and only a few intra-domain connections were observed within Fungi.

## Discussion

This study provides for the first time a deep insight into the microbial biodiversity of biocrusts from coastal sand dunes and the surrounding sands by studying Bacteria and eukaryotic microorganisms at the same time. We detected a lower alpha-diversity of Bacteria and Fungi in biocrust samples compared to the neighboring sand. This observation is in congruence with previous studies from forest soils in mesic regions (Glaser et al., 2022) and from sandy soils in (semi)-arid regions (Abed et al., 2019; Moreira-Grez et al., 2019; Pombubpa et al., 2020), where a lower richness of Bacteria and Fungi was also observed in the respective biocrusts compared to adjacent bare soil. On the contrary, studies from loess plateau in humid regions and from weakly developed soils in arid regions revealed a higher fungal and bacterial richness in biocrusts than in bare soils (Xiao and Veste, 2017; Chilton et al., 2018; Maier et al., 2018). These contrasting results might be explained by microclimatic differences, but would need further investigations in a meta-study comparing worldwide biocrust biodiversity taking into account climatic and edaphic parameters. Despite the richness trend observed for Bacteria and Fungi at the coastal dune sites, the total DNA content as rough proxy for organisms' abundance was higher in biocrusts than in sands throughout all samples. A higher biomass in biocrusts than in the neighboring bare soil has been commonly reported throughout the literature by quantitative PCR or lipid analysis (Steven et al., 2013; Maier et al., 2018; Zhang et al., 2018; Kurth et al., 2020; Ngosong et al., 2020).

The phylogenetic composition of the biocrust microbiome in dunes was similar to that of previously published studies, with Ascomycota (Fungi), Proteo-, Actinobacteria, Oscillatoriophycideae (Cyanobacteria), and Archaeplastida (Eukaryota) as dominant groups (Steven et al., 2014; Xiao and Veste, 2017; Rippin et al., 2018). A significant difference in the community composition between biocrusts and neighboring sand samples was mainly explained by the higher relative abundance of phototrophic organisms: Cyanobacteria and the Archaeplastida were 1.6 times more abundant in biocrusts than in the neighboring sand, with 31% of Cyanobacteria and 67% of Archaeplastida in biocrust samples. A study on biocrusts from polar regions also reported the high relative abundance of Archaeplastida, especially of the microalgal families Chlorophyceae and Zygnematophyceae, similar to our study (Rippin et al., 2018). These data suggest that biocrusts can be regarded as hotspot for biodiversity of phototrophic microorganisms.

Microbial community composition was more similar between sampling sites than between samples of the same habitat (sand or biocrust). This suggests that biocrust microbial community is a subset of the neighboring sand/soil microbiome. This observation is also in congruence with previous studies, where also a significant high similarity was observed between biocrusts and the neighboring soil (Abed et al., 2019; Pombubpa et al., 2020).

For the Eukaryota and cyanobacteria-specific dataset, we observed a similar richness between biocrusts and adjacent sand. An average of 100 cyanobacterial OTUs per site was revealed in biocrusts using cyanobacteria-specific primers, which is in the range reported for other biocrust studies worldwide using the same primers set (Williams et al., 2016; Muñoz-Martín et al., 2018; Roncero-Ramos et al., 2020; Wang et al., 2020; Pushkareva et al., 2021b). Cyanobacteria surrounded by an extracellular matrix, which supports desiccation tolerance, are commonly observed as dominant species in biocrusts from arid and semi-arid regions on organic-poor soils (Büdel et al., 2016). Even though the studied sites are located in a mesic region with regular precipitation all year round, sand dunes might represent a rather dry microhabitat due to very low water-holding capacity of sand and no shading by vascular plants, at least at our sampling sites. Primary sand dunes are an accumulation of sediment without any preliminary soil development, which is reflected by a lack of organic content. Hence, sand dunes represent an organic-poor ecosystem with frequent desiccating conditions (Maun, 2009). In general, such conditions favor nitrogen-fixing Cyanobacteria surrounded by a mucilage sheath, such as members in the *Nostoc* genus. Filamentous Cyanobacteria embedded in a mucilage sheath can potentially initiate biocrust formation by gluing soil particles to a stable biocrust, such as *Microcoleus* spp., which is frequently found and abundant in dunes and in desert biocrusts (Garcia-Pichel et al., 2001; Kidron et al., 2010; Mikhailyuk et al., 2019).

The universal eukaryotic primer set TAReuk was applied to the best of our knowledge only a few times in biocrust research but always without comparison to bare soil. Two studies conducted in polar and sub-polar regions reported equal or lower number of OTUs (average of 485, 242, and 154 OTUs per site in biocrusts of Iceland, the Arctic, and Antarctica) (Rippin et al., 2018; Pushkareva et al., 2021a) compared to our study (480 OTUs per site). Another investigation on biocrusts of extremely saline potash heaps in Germany revealed a lower number of OTUs (average of 61 OTUs per site) (Pushkareva et al., 2021b) compared to our study, indicating that only a few eukaryotic species were able to thrive in this extreme saline habitat. Most OTUs assigned to phototrophic organisms were detected in both biocrusts and adjacent sand, which points toward a rich “seed bank” in the sand surface and its high potential to develop to a biocrust. In addition, lichenicolous Fungi assigned to five genera were detected in biocrust and sand samples alike, although no lichen thallus was visible in either habitat. It might be that fungal spores can be regularly detected as most of the genera are ubiquitous in northern Germany (Weber, 2001; Schiefelbein et al., 2018), which would also contribute to a “seed bank” of photosynthetic organisms, in this case lichens. Similarly, Bryophyta were identified although no moss thalli were visible. In this case, OTUs assigned to Bryophyta were more abundant in biocrusts. It is reasonable to assume that Bryophyte spores already started to form protonema in biocrusts, which is a filamentous thalloid structure that develops into a moss thallus. Additionally, the filamentous streptophytic algae Klebsomidiaceae were detected in high relative abundance (up to 50%) in biocrusts, as well as in the sand, although the sand was free of any visual algal growth. This observation is in congruence with morphological data on coastal sand dunes that also detected members of the genus *Klebsormidium* in high abundance (Schulz et al., 2016; Mikhailyuk et al., 2019). This points toward the importance of filamentous algae for biocrust formation as filaments interweave sand particles and stabilize soil surface by gluing them together due to the excretion of sticky mucilage (Büdel et al., 2016). *Klebsormidium* is known to withstand desiccating conditions, UV radiation, and is characterized by a wide temperate tolerance, which explains its high abundance in sand dunes along the Baltic Sea (Holzinger and Karsten, 2013; Donner et al., 2017; Hartmann et al., 2020).

Microalgae are key players in biocrusts, but high-throughput-sequencing is only occasionally applied to uncover their biodiversity in biocrusts. One reason might be that a primer pair covering all microalgae lineages with similar efficiency and at the same time excluding non-algae protist lineages is missing. Although many terrestrial algae belong to Chlorophyta, terrestrial diatoms and Chrysophyceae are commonly observed (Glaser et al., 2015; Schulz et al., 2016), which represent distinct phylogenetic lineages. The polyphyletic nature of algae is the main obstacle for a primer pair that covers all algae groups but excludes heterotrophic Protists. Using universal primer sets has the disadvantage that those primer sets are often not really “universal” and can miss some phylogenetic lineages (Pawlowski et al., 2012). Further, high copy numbers of the target gene due to multiple nuclei per individual in, for example, metazoans, and ciliates, can bias the relative abundance of OTUs. Specific primer pairs for a group of interest allow a deeper insight into its ecological function. One successful example is the group of Cercozoa, which is abundant and highly diverse in biocrusts, including their feeding habitat (Fiore-Donno et al., 2019; Roshan et al., 2020). Nevertheless, TAReuk primers used in our study covered most algal lineages and are thus a useful tool to uncover algal biodiversity using HTS until more specific primer pairs will be available.

Co-occurrence network analysis revealed five times more co-occurrences in the biocrusts than in the neighboring sand, which could be due to the higher microbial abundance in biocrusts. Although we observed higher richness in neighboring sand than in biocrusts, DNA-based methods do not allow a conclusion on microorganism's activity; thus, some OTUs might be inactive and not involved in potential biotic interactions, which could explain more co-occurrences in biocrusts with a lower richness compared to the neighboring sand. In both habitats, Bacteria dominated the network, which might be due to their high richness and numerous ecological traits such as using a wide variety of carbon sources and the ability to mobilize adsorbed nutrients. In biocrusts, Fungi showed much more co-occurrences than the rest of Eukaryota and Cyanobacteria. This could be interpreted that such potential biotic interactions with Fungi become an important trait during biocrust formation. For example, filamentous Fungi interweave soil particles and can thus increase biocrust stability. Further, saprotrophic Fungi could benefit from the higher biomass in biocrust compared to neighboring soil, thereby increasing the nutrient recycling supported by the Fungi's potential to mobilize micronutrients from minerals (Wei et al., 2012). Another interesting aspect is that Bacteria can benefit from Fungi by using fungal filaments as dispersal vectors to colonize new areas (Deveau et al., 2018). Although phototropic Eukaryota have a higher relative abundance in biocrusts compared to sand, the number of co-occurrences did not increase accordingly. It could mean that biotic interactions with phototrophic organisms, like algivory or nutrient exchange, did not establish yet in the biocrusts from yellow dunes. The protist taxon Cercozoa (SAR supergroup) includes many algivorous species (Fiore-Donno et al., 2019; Dumack et al., 2020) and was found in high abundance in both habitats, indicating the importance of its members as consumers. Previous studies in the same dune habitat confirmed indeed the high abundance of Cercozoa and assigned 20% of all Cercozoa to eukaryvore feeding behavior (Roshan et al., 2020, 2021). Nevertheless, using universal eukaryotic primers, no co-occurrences between algae and potentially algivorous Cercozoa (like members from the families Thecofilosea and Euglyphidae) as potential predator-prey-interactions were observed in higher frequencies in biocrusts than in sand.

## Conclusion

This study provided for the first time a comprehensive insight into biocrusts from coastal dunes, a habitat characterized by organic poor sand as substrate and low water-holding capacity, which regularly leads to desiccation and harsh environmental conditions. Results suggested that biocrust microbiomes are recruited from the neighboring sand communities. Sand samples contain most of the phototrophic organisms found in the neighboring biocrusts, although in low abundance, serving as a “seed bank” for biocrust development. Filamentous algae (like *Klebsormidium* spp.) and filamentous Cyanobacteria (like *Leptolyngbya* spp.) were the dominant phototrophic microorganisms, which points toward the importance of filamentous morphotypes for biocrust development. Furthermore, Fungi exhibited more co-occurrences with other microorganisms in the biocrust than in the neighboring sand samples, which reflects a relevant, but not completely understood ecological role during biocrust development, perhaps in biomass degradation and nutrient recycling. In summary, targeted high-throughput sequencing with different primer sets allows an insight not only in the biodiversity of microorganisms in biocrusts but also could give an idea about potential biotic interactions among different taxa. A combination of structural and functional data based, for example, on lab feeding experiments is essential for a better understanding of biocrust communities in different habitats.

## Data Availability Statement

The datasets presented in this study can be found in online repositories. The names of the repository/repositories and accession number(s) can be found in the article/Supplementary Material.

## Author Contributions

KG and UK developed the idea. AV and KG collected the samples. AV processed and analyzed the samples in the lab. Sequence processing was conducted by IB. Data interpretation and visualization by IB, EP, and KG. KG wrote the first draft of manuscript with contributions by all coauthors. All authors have read and agreed to the published version of the manuscript.

## Funding

Rudolf-and-Helene-Glaser foundation financially supported this research. This research was partly funded by the P-Campus (the Leibniz ScienceCampus Phosphorus Research Rostock).

## Conflict of Interest

The authors declare that the research was conducted in the absence of any commercial or financial relationships that could be construed as a potential conflict of interest.

## Publisher's Note

All claims expressed in this article are solely those of the authors and do not necessarily represent those of their affiliated organizations, or those of the publisher, the editors and the reviewers. Any product that may be evaluated in this article, or claim that may be made by its manufacturer, is not guaranteed or endorsed by the publisher.

## Supplementary Material

Supplementary Figure 1 Analysis of bacterial OTUs comparing biocrust and neighboring sand samples; taxonomic composition of the bacterial community: relative abundance of each clade was compared between biocrust and sand samples (log2 ratio between the median) and indicated with colors. Color code is given below the figure (basically Figure 3A with all taxonomic affiliation).

Supplementary Figure 2 Analysis of eukaryotic OTUs comparing biocrust and neighboring sand samples; taxonomic composition of the eukaryotic community: relative abundance of each clade was compared between biocrust and sand samples (log2 ratio between the median) and indicated with colors. Color code is given below the figure (basically Figure 5A with all taxonomic affiliation).

Supplementary Figure 3 Analysis of fungal OTUs comparing biocrust and neighboring sand samples; taxonomic composition of the fungal community: relative abundance of each clade was compared between biocrust and sand samples (log2 ratio between the median) and indicated with colors. Color code is given below the figure (basically Figure 6A with all taxonomic affiliation).

Supplementary Table 1 List of primers used in this study.

Supplementary Table 2 Observed co-occurrences in biocrusts from sand dunes resolved on phylum level for all domains; extended Information to Figure 8.

Supplementary Table 3 Observed co-occurrences in neighboring sand resolved on phylum level for all domains; extended Information to Figure 8.
