# Supplementary figures and images for: Microbial Communities in Biocrusts Are Recruited From the Neighboring Sand at Coastal Dunes Along the Baltic Sea
Source: Front Microbiol. 2022 Jun 16;13:859447. doi: 10.3389/fmicb.2022.859447 (PMC9245595; doi:10.3389/fmicb.2022.859447)

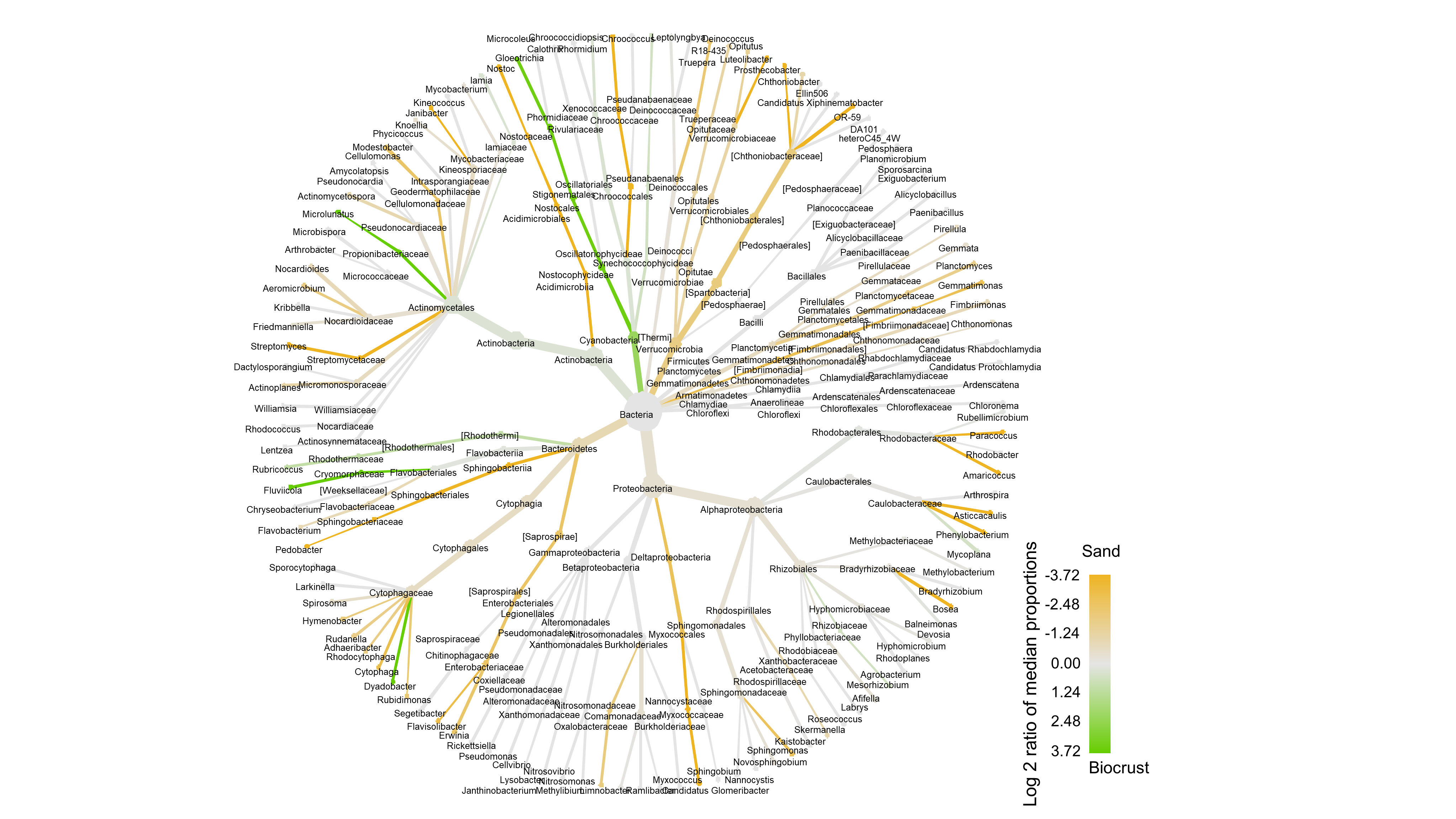

Supplement: Supplementary Figure 1 — Analysis of bacterial OTUs comparing biocrust and neighboring sand samples; taxonomic composition of the bacterial community: relative abundance of each clade was compared between biocrust and sand samples (log2 ratio between the median) and indicated with colors. Color code is given below the figure (basically Figure 3A with all taxonomic affiliation). [file Image_1.TIF]

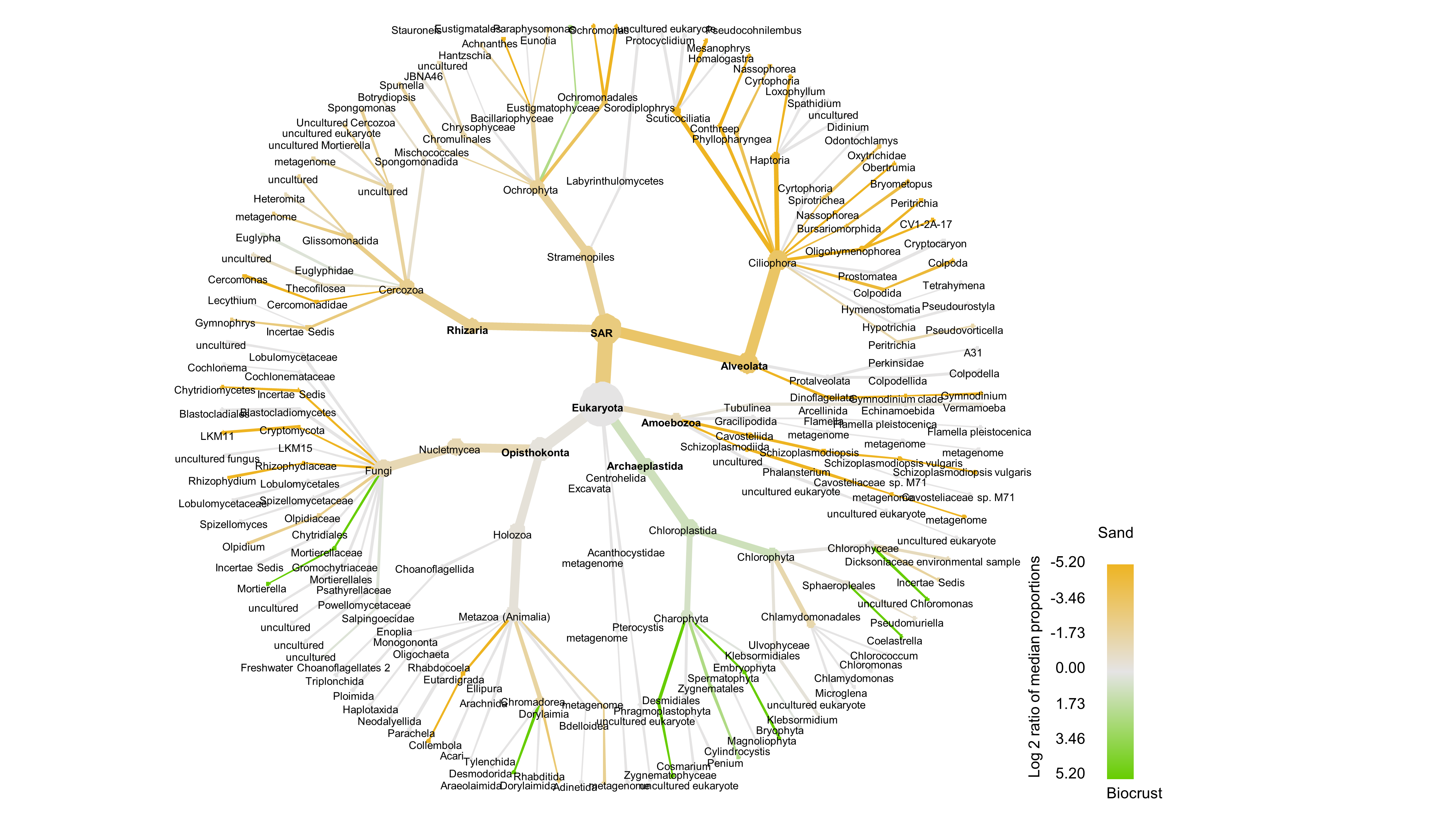

Supplement: Supplementary Figure 2 — Analysis of eukaryotic OTUs comparing biocrust and neighboring sand samples; taxonomic composition of the eukaryotic community: relative abundance of each clade was compared between biocrust and sand samples (log2 ratio between the median) and indicated with colors. Color code is given below the figure (basically Figure 5A with all taxonomic affiliation). [file Image_2.TIF]

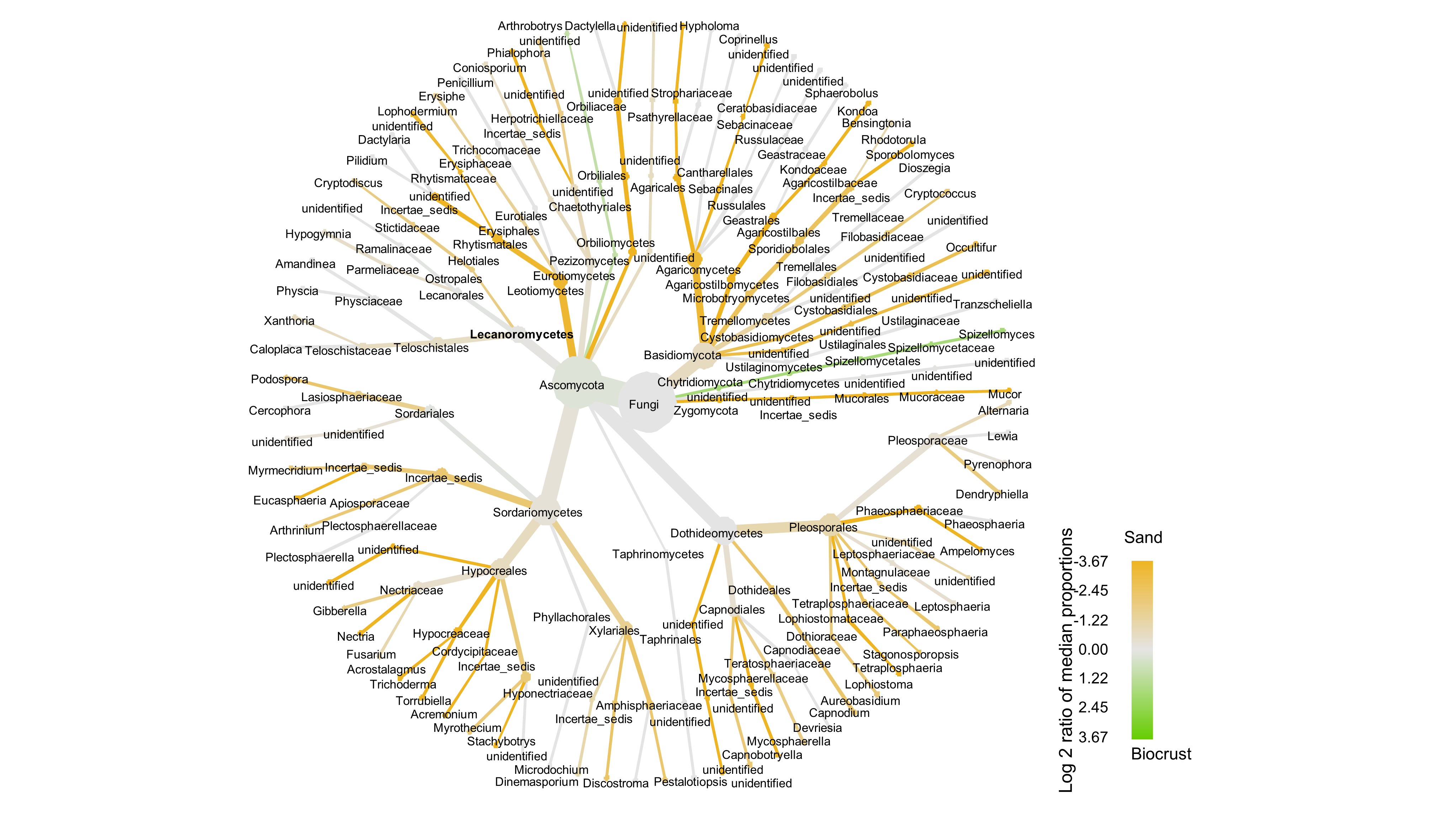

Supplement: Supplementary Figure 3 — Analysis of fungal OTUs comparing biocrust and neighboring sand samples; taxonomic composition of the fungal community: relative abundance of each clade was compared between biocrust and sand samples (log2 ratio between the median) and indicated with colors. Color code is given below the figure (basically Figure 6A with all taxonomic affiliation). [file Image_3.TIF]
